# Editorial: Functional surfaces and biomaterials

**DOI:** 10.3389/fbioe.2023.1138172

**Published:** 2023-01-17

**Authors:** Yuangang Liu, Rumen Krastev, Junchao Wei, Min Jiang

**Affiliations:** ^1^ College of Chemical Engineering, Huaqiao University, Xiamen, China; ^2^ Faculty for Life Sciences, Reutlingen University, Reutlingen, Germany; ^3^ School of Stomatology, Nanchang University, Nanchang, China; ^4^ School of Biotechnology and Pharmaceutical Engineering, Nanjing Tech University, Nanjing, China

**Keywords:** surface modification, coating, cell-material interaction, functionalized materials, functionalized surfaces, drug delivery systems, immobilization of biomolecules, miniaturized biosensor and bioreactors

## Functional surfaces and biomaterials

At the beginning of 2022, Frontiers in Bioengineering and Biotechnology - Biomaterials Section has published a Research Topic on “Functional Surfaces and Biomaterials.” The aim of this Research Topic is to summarize the current state of research and development in the field of functional surfaces and biomaterials with a particular focus on biotechnological and medical applications.

The guest editorial team would like to thank all colleagues from around the world who submitted their reviews and research articles for the Research Topic. By the end of August 2022, we have successfully collected 20 articles by 138 participating authors following the peer review process. We also tried to select manuscripts from different research areas to cover the most relevant Research Topic of interest, from drug delivery systems to bone tissue engineering to biosensors and general aspects in biomedicine. By the end of December, the 20 articles had been viewed for more than 21000 times with downloads more than 4,000 times, and 11 articles have reached more than 1,000 views.

Among the 20 articles, the interests mainly focus on tissue engineering, especially bone tissue engineering, including the influence of divalent cations on osteogenic mechanism (L. Fan et al.; X. Nie et al.), the modification of materials on cell response and osteogenesis promotion (F. Guo et al., Z. Zheng et al.), and the antibacterial coating on the surface of intraosseous implants (X. Bai et al.). For example, L. Fan et al. described the synergistic effects on osteogenesis and angiogenesis by the controlled release of Ca^2+^, Mg^2+^, and Cu^2+^ ions. The simple technique used in this article served to address the adverse effects of using biologics in bone tissue engineering, the availability of which is quite limited due to regulatory issues. In addition, the osseointegration of tianium dental implants also has been investigated by N. López-Valverde et al., A. López-Valverde et al. and J. Aragoneses et al., which could be of general interest as titanium oxide-based materials are widely used in orthopedic treatments. The article by L. Guo et al., reported a biomimetic hepatic lobule-like model that could be a robust platform for various medical applications. The article by Y. Shan et al. reported modification of ePTFE with heparin/collagen-REDV can promote the cytocompatibility and antiplatelet property.

Drug delivery system is another intensive field. Researchers prepared carriers with different modes such as stimuli-responsive nanocarriers with transformable size for multistage drug delivery (Z. Liu et al.). The contribution from P. Wang et al. described CaCO_3_ nanorods for tumor therapy demonstrates a possible rethinking of the use of fundamental and widely studied materials to address medical challenges. Gas–involved chemosensitization strategy is also proposed for cancer treatment. For example, L. Tian et al. prepared a tumor-specific lipase-responsive nanomedicine based on aptamer-conjugated DATS/Dox co-loaded PCL-b-PEO micelle for pancreatic cancer, F. Wang et al. reported magnetic resonance imaging-guided low-frequency focused ultrasound combined with GDNF microbubbles was used to target BBB opening in the ventral tegmental area (VTA) region. New materials such as Hagfish proteins (R. Sun et al.), and phase-change materials (B. Chen et al.), as well as diatom biosilica label-free biosensor (T. Chen et al.), and bionic neural interfaces (J. Zhang et al.), are also reported in this Research Topic.

An interesting work is about cell expansion and microtissue construction. S. Wu et al. developed a unique magnetic peptide-grafted sorting microsphere to obtain relatively pure and high-yield MSCs in an economical and effective way, which can also be used for the expansion of MSCs. (R. Long et al.) introduced a novel tissue construction strategy using 3D cell culture based on artificial cells and hydrogel under microgravity for bottom-up microtissue constructs, which is beneficial for cell distribution and of great significance for tissue construction research *in vitro*.

In summary, given their importance, it is perhaps not surprising that the functional surfaces and functionalization using biomaterials are highly interesting areas in the biomedicine. The main reasons are the cellular behavior and the reactions to differently structured and chemically presented surfaces. To control these effects and to use them for the improvement of clinical outcomes, research and development in the fields of materials, process engineering and analytical strategies are required. With the new trend of circular economy, resource efficiency and value chain becoming a driving force for innovation, active collaboration between researchers, clinicians and industry is essential and increasingly important. The close collaboration was also evident in the research articles, as various laboratories and hospitals were engaged in the contributed papers. It is anticipated that the better understanding of the functional surfaces and biomaterials will promote the applications of biomaterials in the future.

